# MicroRNA profiling during directed differentiation of cortical interneurons from human‐induced pluripotent stem cells

**DOI:** 10.1002/2211-5463.12377

**Published:** 2018-02-17

**Authors:** Jiajie Tu, Dandan Cao, Lu Li, Hoi‐Hung Cheung, Wai‐Yee Chan

**Affiliations:** ^1^ Ministry of Education Key Laboratory for Regenerative Medicine (CUHK‐Jinan University) School of Biomedical Sciences Faculty of Medicine The Chinese University of Hong Kong Hong Kong SAR China; ^2^ CUHK‐CAS Guangzhou Institute of Biomedicine and Health Joint Laboratory on Stem Cell and Regenerative Medicine School of Biomedical Sciences Faculty of Medicine The Chinese University of Hong Kong Hong Kong SAR China

**Keywords:** cortical interneurons, human iPSCs, microRNA

## Abstract

Induced pluripotent stem cells (iPSCs) are useful for modeling neuron development and related diseases. Cortical interneurons are essential players in neuropsychiatric diseases such as autism. miRNAs are a class of pivotal regulators in neural differentiation. Using a previously established model of cortical interneuron differentiation from human embryonic stem cells, we profiled miRNAs involved in differentiation from human iPSCs. A number of miRNAs were modulated in the differentiation process. This study captured the temporal *in vitro* neurogenesis from iPSCs to mature cortical interneurons. The specific miRNAs identified at each stage of differentiation are of potential use for drug discovery and prospective clinical applications.

**Abbreviations**
CNS: central nervous system
ESCs: embryonic stem cell
iPSCs: induced pluripotent stem cells
SHH: sonic hedgehog

Human‐induced pluripotent stem cells (iPSCs) are an useful platform for modeling human diseases. Due to the lack of effective therapies for many neurodegenerative disorders, iPSC differentiated toward neural lineages of the central nervous system (CNS) is very promising. Early related studies of using iPSCs in neurodegenerative diseases include midbrain dopamine neurons in Parkinson's disease (PD) 1, motor neurons in amyotrophic lateral sclerosis (ALS) 2 and spinal muscular atrophy (SMA) 3. Recent studies show the possibility of treating some other neuronal‐related dysfunctions with iPSC‐derived neuronal cells, such as schizophrenia 4 or autism 5. However, the neuron subtypes critical for modeling schizophrenia or autism are still not well established. Protocol for the derivation of embryonic stem cell (ESC)‐derived cortical projection neurons has been established 6, while the other essential neuron subtypes, such as cortical interneurons, which are involved in schizophrenia or autism, are needed to model these neurological disorder 7. Recently, differentiation of cortical interneurons from ESC has been reported 8.

Here, we validate the efficient differentiation of human cortical interneurons from human iPSCs *in vitro*. It demonstrates that the specific differentiation of distinct human cortical interneuron populations from iPSCs could be achieved by combining tankyrase inhibitor XAV939, sonic hedgehog (SHH) recombinant protein, BMP inhibitor LDN193189, inhibitor of activin receptor‐like kinase SB431542 and SHH activator purmorphamine treatment. However, how to efficiently apply iPSC‐derived cortical interneuron into clinical application is still a big challenge, which requires a more comprehensive elucidation of the molecular regulation involved in directing the differentiation.

miRNAs are a group of master regulators in balancing between stem cell self‐renewal and neural lineage differentiation 9. However, little knowledge is known about the role of miRNA as potential key regulators of neurogenesis during cortical interneuron differentiation. In the current study, we investigated global miRNA profile using the platform of iPSCs differentiation into cortical interneurons. Taking advantage of miRNA sequencing analysis, we demonstrated the distinct miRNA profiles during the differentiation process from undifferentiated iPSCs to mature cortical interneurons. This study has expanded our understanding of the role of miRNA during cortical interneuron differentiation and maturation.

## Materials and methods

### Cortical interneuron differentiation from iPSCs

**Figure 1:**
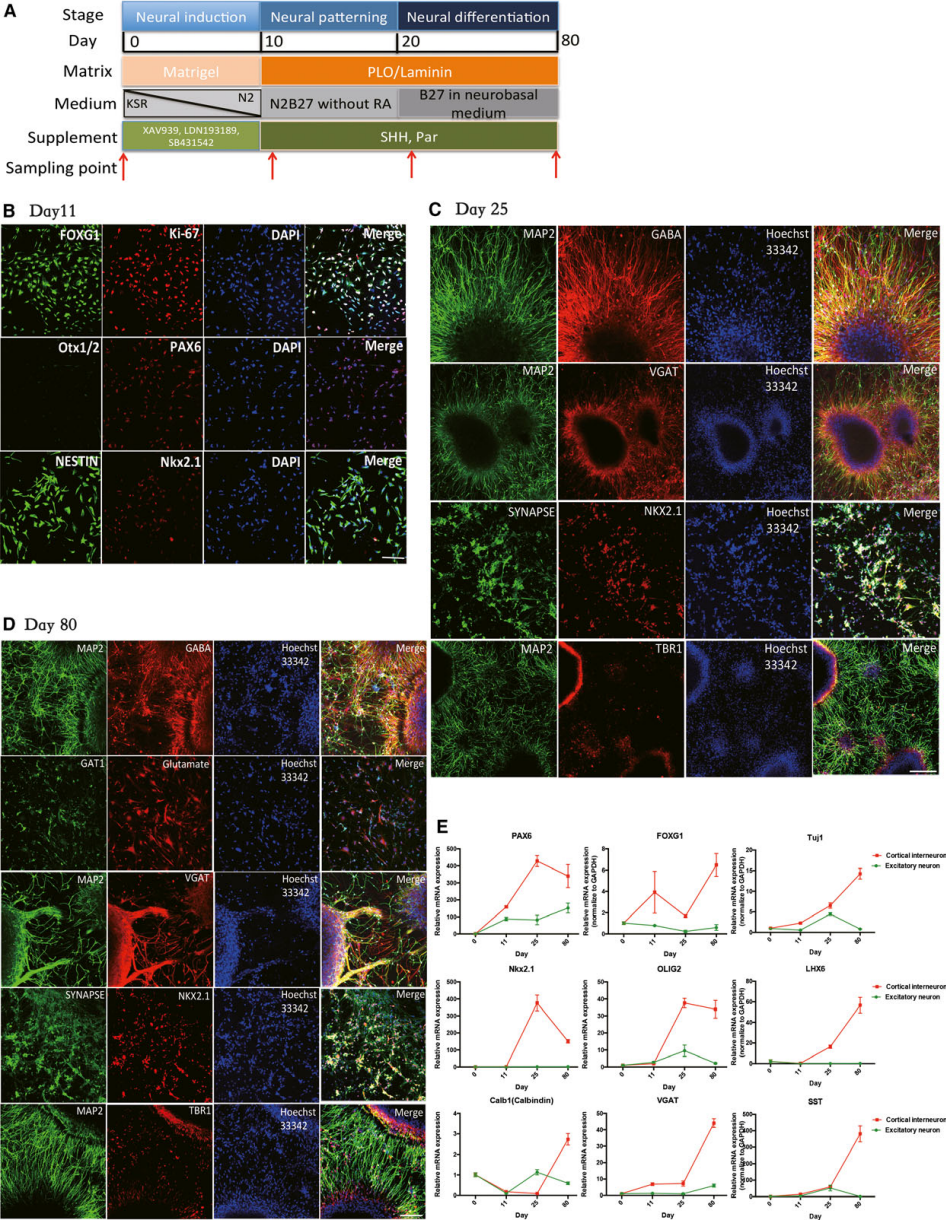
Directed differentiation of cortical interneuron from human iPSCs. (A) Schematic illustration of three stages of cortical interneuron differentiation; (B–D) Immunofluorescence for neuronal markers at precursor, immature and mature stages of cortical interneuron differentiation; the scale bar represents 100 μm; (E) qPCR for specific neuron markers during differentiations of excitatory and cortical interneurons.

Human iPSCs were maintained on mouse embryonic fibroblasts and dissociated with accutase for differentiation or dispase for passaging. The source of iPSCs is iBC‐1.2, which is a normal iPSC line generated at our own lab. It was derived from dermal fibroblast of a male healthy donor (age 32). The iPSC line has been characterized for pluripotency and normal karyotype 10. Differentiation media [knockout serum replacer (KSR) + N2 medium for neural induction, N2 + B27 (GIBCO) for neural patterning and neurobasal medium + B27 for neuronal differentiation] were described previously 8 and are shown in Fig. 1A. The involved compounds include XAV939 (2 mm; Stemgent, Lexington, MA, USA), LDN193189 (100 nm; Stemgent), SB431542 (10 mm; Tocris Bioscience, Avonmouth, Bristol, UK), purmorphamine (2 mm; Calbiochem, Darmstadt, Germany) and Recombinant SHH (C25II; 500 ng·mL^−1^).

### Immunofluorescence

Cells (~ 1 × 10^5^) were cultured on a cover glass in a 12‐well plate with 700 μL of medium. The neural cells were allowed to grow to desired morphology and density before staining procedure. Cells were first washed once with PBS and fixed by 4% paraformaldehyde/4% sucrose in PBS at room temp, followed by permeabilization and DNA denaturation by 0.2% TritonX‐100 in 4 m HCl. After that, the cells were washed with PBS and blocked in 80 μL BSA (3%). The cells were incubated with anti‐FOXG1 (ab18259; Abcam), Ki‐67 (BD, 550609), Otx1/2 (ab21990; Abcam, Cambridge, MA, USA), PAX6 (ab195045; Abcam), NESTIN (BD, 561230), Nkx2.1 (MAB5460; Millipore, Darmstadt, Germany), MAP2 (M4403; Sigma, St. Louis, MO, USA), GABA (A2052; Sigma) VGAT (131011; Synaptic systems, Goettingen, Germany), SYNAPSIN (Abcam), TBR1 (ab31940; Abcam), GAT1 and Glutamate (ab1511; Millipore) in BSA (3%) at 4 °C overnight, and then conjugated with and Hoechst 33342 or DAPI. The glass slides were mounted with a cover slip before imaging.

### RT‐qPCR

Total RNA was extracted by Trizol reagent (Invitrogen, Waltham, MA, USA) according to standard protocol. The concentration and quality of all RNA samples were evaluated by Nanodrop 2000 (Thermo, Waltham, MA, USA), and the 260/280 and 260/230 values of all samples were above 1.8 and 1.9, respectively. Gene reverse transcription was performed with a MasterMix kit (Takara, Mountain View, CA, USA) and miRNAs reverse transcription was performed with a TaqMan reverse transcription kit (Life technology, Waltham, MA, USA) following the standard manuals. Quantitative PCR of gene was performed using a Universal SYBR Green Master mix (Applied Biosystems, Waltham, MA, USA) and miRNAs qPCR was performed using a TaqMan specific miRNAs probe (Life technology) on a StepOnePlus real‐time PCR system (Applied Biosystems). Gene expression was normalized to Gapdh and miRNAs expression was normalized to U6 unless otherwise stated.

### Electrophysiology

Microelectrode arrays (MEAs) recordings were performed as described previously 11.

### miRNA profiling

Total RNA was isolated for small RNA library construction and further to be next generation deep sequenced. The sequencing tags were aligned by bowtie 2 12. Known miRNAs were analyzed by mapping to miRNA sequences from mirbase V21 13. Novel miRNA were predicted using mirdeep2 14. Based on the known miRNA and novel miRNA sequence information, miRNA expression were calculated by counting the number of tags mapped to the miRNA, and finally, normalized to tags per million (TPM).

### Time point specificity index (TSI)

To evaluate the expression pattern during cortical interneuronal differentiation, we calculated a time point specificity index (TSI) for each miRNA similar to 15. This index is a measurement for the miRNA expression specificity regarding to different time points. TSI value ranges from 0 to 1, with value close to 0 representing miRNAs expressed in all time points and value of 1 representing miRNAs expressed in only one time point. The TSI for a miRNA *j* is calculated as

$$\text{TSI}j=\frac{\sum_{i=1}^{N}\left(1-xj,i\right)}{N-1}$$

where *N* corresponds to the total number of time points measured and *xj, i* is the TPM value of time point *i* normalized by the maximal TPM of any time point for miRNA *j*. The reason to select miRNA families containing at least five mature miRNAs is because five is an appropriate number to examine TSI distribution within one family 15.

### Differential expression analysis of miRNAs

To detect differentially expressed miRNAs during cortical interneuronal differentiation, we compared D11, D25 and D80 miRNomes to D0 miRome. An r package named DEGseq 16 was used which assume a binominal distribution model of read count (not TPM) for each miRNA to calculate the *P*‐values and fold change. Calculated *P*‐value < 0.01 and fold change larger than 2 were set as the cutoff to identify significantly differentially expressed miRNAs.

### Clustering of miRNA expression

Normally, miRNAs with similar expression patterns usually have functional correlation. To display the clustering information of defined set of miRNAs, heatmap analysis based on the miRNA expression value was conducted. While for the sample clustering, we took all miRNA TPM as input and used hcust in r to get the dendrogram.

### Search for target genes of miRNAs and for miRNAs of given genes

Known human miRNA‐target interactions (MTI) were downloaded from the miRTarBase 17 database (http://mirtarbase.mbc.nctu.edu.tw/php/download.php; cataloged as hsa_MTI.xlsx; accessed on 10/07/2017). List of the targets of downregulating and upregulating known miRNAs were retrieved taking miRNA names as the key. List of miRNAs that target given genes were obtained taking gene names as the key.

### KEGG and GO enrichment analysis

Selective list of genes found to be targeted by downregulating/upregulating miRNAs were subjected to KEGG pathway and GO enrichment analysis using david 6.8 online tools 18 (https://david.ncifcrf.gov/home.jsp) with default parameters. All human genes were used as the background.

## Results

### Directed differentiation of human iPSCs to cortical interneuron

Cortical interneurons were differentiated from iPSCs through three stages, namely neural induction, neural patterning and neural differentiation, according to the previous report 8 (Fig. 1A). Approximately 10 days after neural induction, neural rosette‐like structure was formed, resembling the early neural tube (Fig. S1A). The numerous clusters of columnar cell‐formed rosettes were manually picked to eliminate contamination from other non‐neural cells.

Immunofluorescence showed that the derived neuron progenitors were positive for the progenitor markers Nestin. Precursor markers FOXG1 (forebrain marker) and PAX6 were also expressed (Fig. 1B). Proliferation marker Ki‐67 was also highly expressed at this time point. Forebrian progenitor marker NKX2.1 began to express while OTX1/2 (general anterior marker) did not show up. Under the defined culture conditions with specific supplements SHH and Par, neural progenitor could be cultured up to 80 days, consistent with a previous report 19. After 12 days of neural patterning, neural progenitor gave rise to neurons positive for NKX2.1, among which some expressed the precursor of cortical neuron markers, such as GABA (cortical interneural marker), MAP2, TBR1 and also the neural synapse marker SYNAPSE (Fig. 1C). The last stage is to induce precursor to mature cortical interneurons (termed as ‘neural differentiation’) for up to 80 days. The specific cortical interneural markers, such as TBR1, GABA, MAP2, GAT, VGAT, glutamine, were all highly expressed, suggesting the differentiation efficiency is robust (Fig. 1D). In addition, spontaneous firing was detected at day 80 (Fig. S1B) and several markers were also compared between excitatory and cortical interneuronal differentiation by qPCR (Fig. 1E), which further confirmed the specificity of our differentiation condition.

### Distribution of miRNAs during cortical interneuronal differentiation from hiPSCs

**Figure 2:**
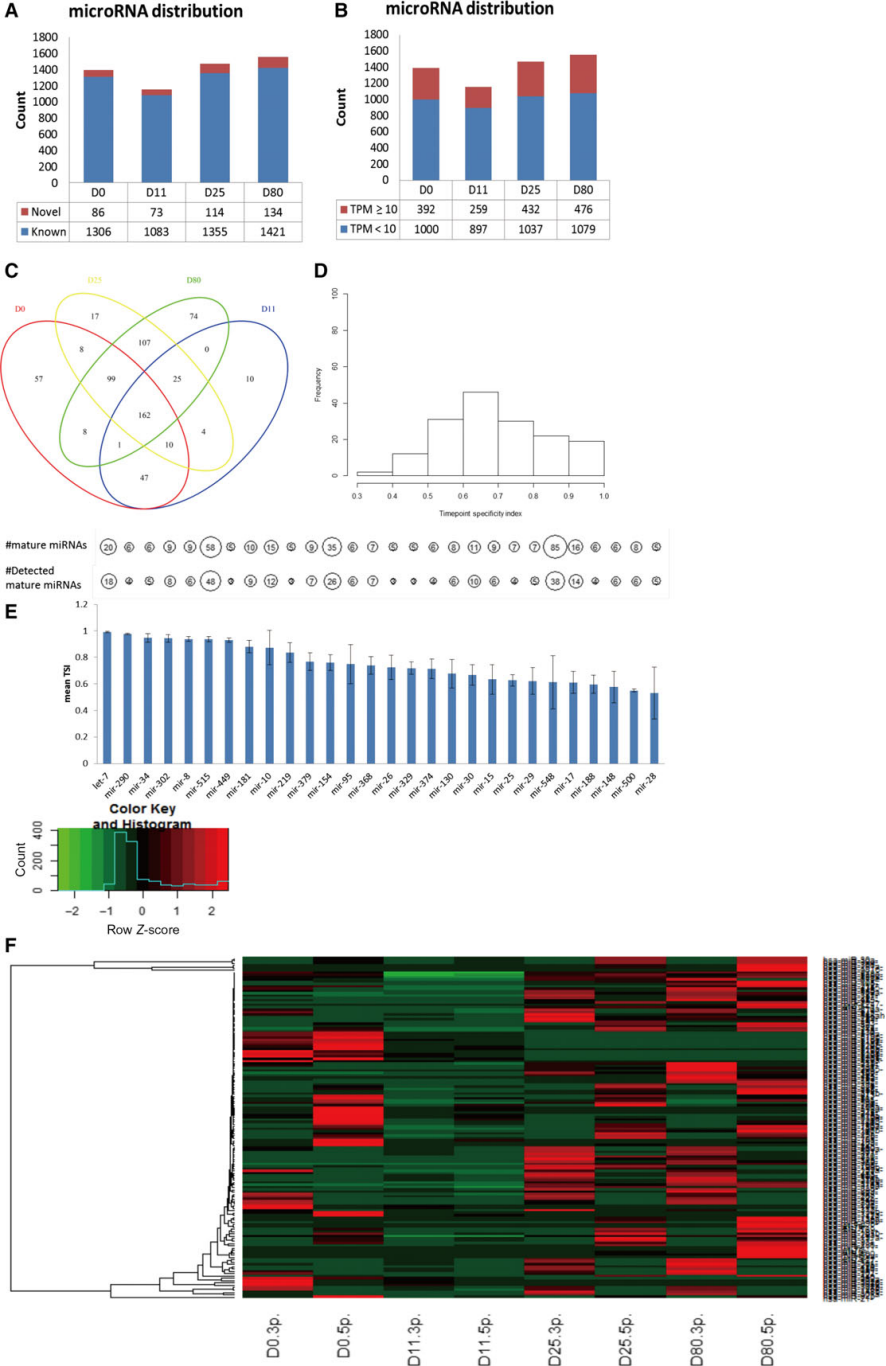
Characteristics of miRNAs during cortical interneuron differentiation. (A) Distribution of known and novel miRNAs detected in each sample; (B) Distribution of miRNAs under defined TPM cutoff in each sample; (C) Venn diagram of miRNAs with TPM ≥ 10 in each sample; (D) Time point specificity (TSI) distribution of miRNAs detected in all samples; (E) Barplot of TSI value (mean ± SD) in miRNA families which contain at least five members and at least three out of which were detected (TPM ≥ 10) in our dataset. Number of miRNA members and number of detected miRNA members in each miRNA family were indicated in top circles; (F) Heatmap for the 146 pairs of miRNAs with ‐3p and ‐5p mature forms at each time point. Normalized TPM values were used.

To delineate the dynamic change of miRNAs along cortical interneuronal differentiation, we performed deep miRNA sequencing to reveal the miRNome across four time points (D0, D11, D25, D80) representing hiPSCs, neuron progenitor cells, immature neurons and mature neurons, respectively. The read count was normalized to TPM for each miRNA. Thus, we could compare the same miRNA expression across different time points. In total, without considering TPM cutoff, there were 1787 known miRNAs and 270 novel miRNAs detected in at least one time point (Table S1). However, when we set the TPM ≥ 10 (Fig. S1C) as the cutoff for a miRNA to be detected, the number of known miRNAs dramatically dropped to 587, and novel miRNA dropped to 42 (Table S1). The number of miRNAs detected in individual samples distributed evenly as shown in Fig. 2A,B. Based on our filtered dataset, we firstly constructed the dendrogram of our differentiation samples (Fig. S1D). It showed that D0 and D11 samples clustered together while D25 and D80 samples clustered together. This result indicated that the generated miRNomes are representative of distinct stages of neuronal differentiation. By intersecting the miRNAs in each sample (Fig. 2C), we found that most of the miRNAs were widely expressed in more than one samples. To present more comprehensive information on the temporal distribution of miRNAs, we applied the miRNA TSI (time point specific index; 0–1) 15 in our dataset. We calculated TSI for all miRNAs (Fig. S1E) and TSI for 162 miRNAs expressed in all four samples (Fig. 2D). It was found that although the 162 miRNAs were expressed in all four samples based on TPM cutoff, they still had significant expression level difference across samples (wide TSI distribution). We further examined the miRNAs with TSI score of 1 (Table S1). Most of them were specifically expressed at one time point with TPM lower than 100 which is a relatively small value when compared to the abundant TPM as high as thousands or even more.

Utilizing our dataset, we further determined the extent to which miRNA families showed similar abundances at specific stage of neuronal differentiation by calculating TSI for mature miRNAs inside each family. Out of 589 miRNA families from the miRBase V21 analyzed 20, 37 miRNA families with at least five mature miRNAs were found (Table S1). The reason to select miRNA families containing at least five mature miRNAs is because five is an appropriate number to examine TSI distribution within one family and it is also a parameter described in another paper 15. Among these 37 miRNA families, nine families were excluded since < 3 mature miRNAs of each family were detected in our dataset. As a result, 28 miRNA families were included in our analysis (Fig. 2E). We found that family let‐7 has the highest TSI value. Most members of this family showed stage‐specific abundance in neurons (Fig. S2A). Similarly, the miR‐302 family, with members including ‐3p and ‐5p mature forms of a/b/c/d isoforms was specifically highly expressed in hiPSCs stage (Fig. S2B). Interestingly, some members in these miRNA families are involved in neuronal function or hiPSCs/ESCs pluripotency 21, 22, 23, 24. It suggests that the expression abundance together with the TSI value could be an important indicator for a miRNA to possibly be functioning in the corresponding stage during the differentiation process.

We also queried whether the ‐3p and ‐5p mature forms of miRNAs were coexpressed during neuronal differentiation process. To limit the biases of miRNAs that are annotated with only one mature form, we only included miRNAs with two mature forms. By analyzing 146 pairs of miRNAs, we found that the ‐3p and ‐5p forms were not always consistently expressed during neuronal differentiation (Fig. 2F, Table S1). At D0, D25 and D80, some of them were ‐5p biased expressed, while some of them were ‐3p biased expressed, and some of them are equally expressed (Fig. 2F and Fig. S2C–D). However, at D11, the consistency between ‐3p and ‐5p was high. This phenomenon is interesting. It indicated one possibility that when the balance between ‐3p and ‐5p is affected, abnormal developmental process might happen. However, the mechanism is currently unknown for how ‐3p and ‐5p are selected to be expressed. Further research on this observation is required.

### Differential expression of miRNAs during cortical interneuronal differentiation

**Figure 3:**
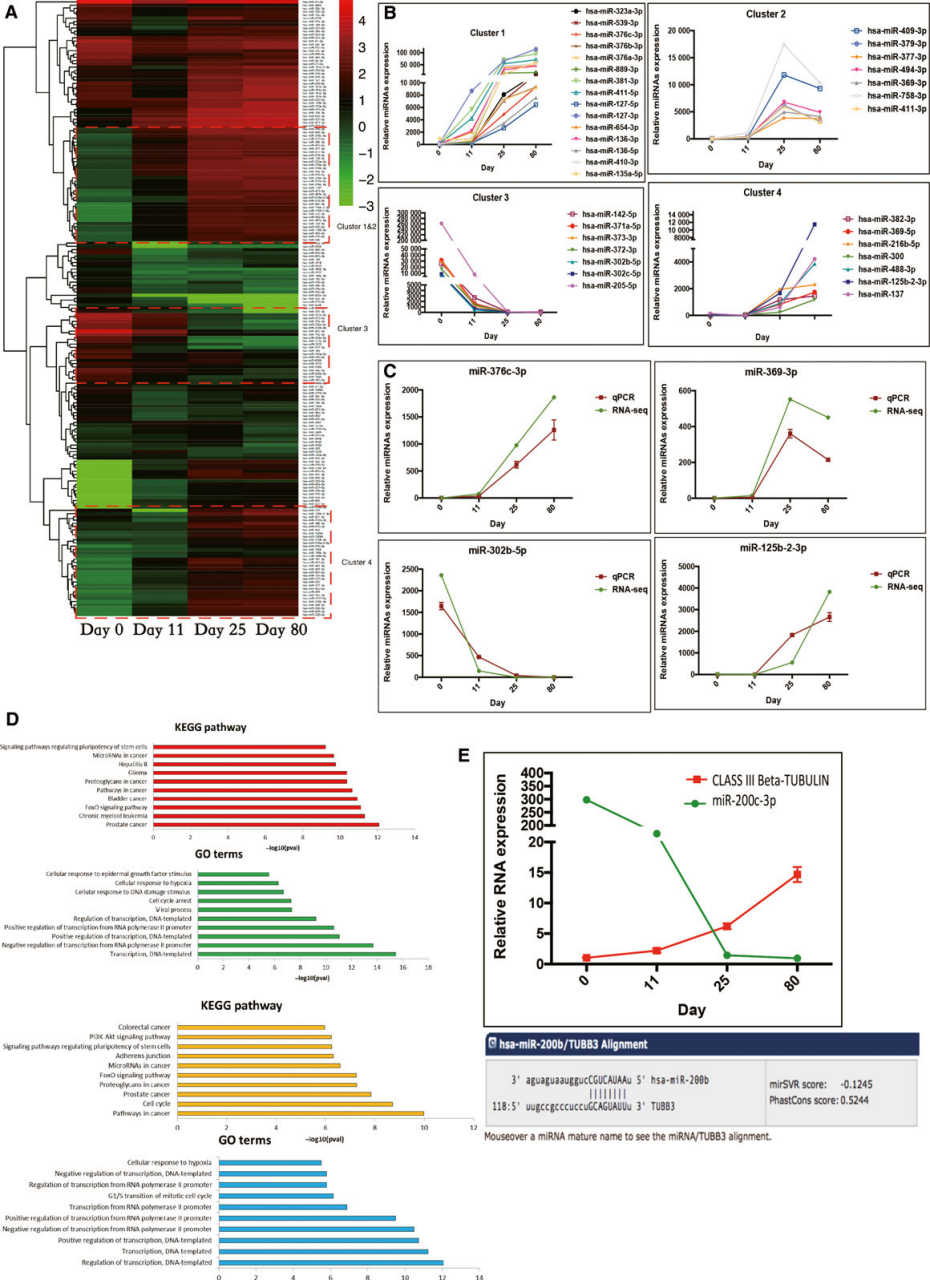
Dynamics of miRNAs during directed differentiation of cortical interneuron. (A) Clustering of miRNAs during directed differentiation of cortical interneuron; (B) Four clusters of miRNAs show different dynamic expression patterns; (C) qPCR validation for typical individual miRNA from four clusters; (D) GO analysis of upregulated and downregulated miRNAs during differentiation of cortical interneuron; (E) Opposite expression patterns and potential interaction of miR‐200c and CLASS III Beta‐TUBULIN during differentiation of cortical interneuron.

To reveal differentially expressed miRNAs during the cortical interneuronal differentiation, we compared D11, D25 and D80 to D0, respectively, using the method described as in the Methods section. Nonredundantly, there were 552 miRNAs found to be differentially expressed during the differentiation process (Table S2). From the heatmap of these miRNAs shown in Fig. 3A, they fell into distinct clusters. Examples from each cluster were shown (Fig. 3B). The Taqman‐based qPCR quantification of miR‐376c‐3p, miR‐369‐3p, miR‐302b‐5p and miR‐125b‐2‐3p was consistent with the sequencing result (Fig. 3C).

In order to narrow down the differentially expressed miRNA candidates important for cortical interneuronal differentiation, we set more stringent criteria. For downregulating miRNAs, miRNAs met the following criteria were included: (a) significantly downregulated at two or more time points with fold change larger than 6.5 when compared to D0; (b) TPM for miRNAs at D0 is larger than 100; (c) TSI value for miRNAs is larger than 0.8. For upregulating miRNAs, first, we only considered miRNAs expressed in another dataset which ensures the miRNAs included are brain‐expressed rather than individual heterogeneity by applying an external dataset 25. Second, miRNAs were retained if they were significantly upregulated at one time point with fold change larger than 6.5 as well as with TPM larger than 100 at one time point. Applying these criteria, we generated miRNA candidate list including 58 downregulated miRNAs and 98 upregulated miRNAs (Table S2). Among them, the functional role of some known miRNAs has been proven in iPSCs such as, miR‐302 family 26, miR‐372 27 and miR‐367 28. On the other hand, the well‐known brain‐enriched miRNAs such as the let‐7 family 29, miR‐124 30 and miR‐9 31 were also found in the list.

Based on the downregulating and upregulating miRNA candidate list, we further found their downstream targets using information from the miRTarBase database to reveal their functions. In total, 7419 targets were found for the 58 downregulated miRNAs (Table S2), while 4471 targets for the 92 upregulated miRNAs (Table S2). In further *in silico* functional analysis, we only considered predicted genes targeted by more than two miRNAs. Selectively, 2010 gene from the downregulating miRNAs and 1472 genes from the upregulating miRNAs were analyzed with the DAVID web tool 18 to identify the associated GO terms and KEGG pathways (Table S3). The top‐enriched KEGG pathways and GO terms were shown in Fig. 3D. Most of the target genes were associated with cancer pathways, stem cell pluripotency regulatory pathways, FoxO signaling pathway and neurotrophin signaling pathway. The obtained results suggest that these differentially expressed miRNAs play a substantial role in the regulation of pluripotency and the neuronal differentiation.

In addition to the global analysis on the downstream targets of the differentially expressed miRNAs during neuronal differentiation, we also utilized our miRNome data to identify upstream miRNAs that potentially target genes known to be involved in cortical interneuronal differentiation. Using miRTarBase database 17, miRNAs that targeted PAX6, FOXG1, CLASS III Beta‐TUBULIN, NKX2.1, NKX6.2, OLIG1, LHX6, CALB1, VGAT, SST and MASH1 were found (Table S4). As an example, the inverse expression of miR‐200c and CLASS III Beta‐TUBULIN indicated hsa‐miR‐200c‐3p might play an important role in cortical interneuron differentiation by repressing CLASS III Beta‐TUBULIN expression (Fig. 3E).

## Discussion

Modeling human psychiatric disease such as autism mainly focuses on using patient‐specific iPSC‐derived neurons 4, 5. However, the *in vitro* differentiation culture usually contains different neuronal subtypes, which complicate the analysis. Therefore, for modeling human disease, it is always important to obtain pure and mature cortical interneurons. The current study shows that the mature cortical interneurons could be differentiated from iPSCs *in vitro*, which could be used as a powerful platform for understanding mechanism leading to the dysfunction of cortical interneuron and prescreening of drugs for neurodegenerative diseases, such as schizophrenia and autism, *in vitro*.

miRNAs are proven to be involved in CNS development. To understand the role of miRNAs from iPSCs to cortical interneuron, we have compared the expression profiles of miRNAs during this process. miRNA profiling for each stage was shown in our results. The significant changes of well‐studied miRNA clusters in iPSCs, such as miR‐302 family 26, miR‐372 27 and miR‐367 28 were included. The well‐known brain‐enriched miRNAs such as the let‐7 family 29, miR‐124 30 and miR‐9 31 also showed neuron‐specific expression.

Most previous neuronal differentiation gave rise to a mixed population, which is a main paradigm for future potential application. Several groups of miRNAs were divided into different groups at different stage of the differentiation process including iPSCs, neuronal progenitor, immature and mature cortical interneuron. Due to multitargeting property of miRNAs, the dynamic expression patterns of miRNAs indicate a complex regulatory network during differentiation of cortical interneuron.

MiR‐200c is one of the miRNAs functioning in cell proliferation 32, 33. It was also reported to promote iPSCs reprogramming and several other lineages differentiation including neurogenesis 34, 35, 36, 37, 38. Consistently, high expression of miR‐200c in iPSCs was observed in our results. Based on the bioinformatics analysis, CLASS III Beta‐TUBULIN was predicted as a potential direct target of miR‐200c, which implies that miR‐200c might directly interfere with the expression of CLASS III Beta‐TUBULIN. Furthermore, the inverse expression between miR‐200c and CLASS III Beta‐TUBULIN indicates that miR‐200c may suppress the expression of CLASS III Beta‐TUBULIN. It is also interesting to note that the changes of miR‐200c during differentiation of mouse primary cortical neurons were consistent with our findings 35. However, the specific role of miR‐200c in cortical interneuronal differentiation definitely needs further investigation.

Epigenetic modification greatly affects neural differentiation from iPSC 39, 40. Other studies demonstrate that miR‐200 family is tightly regulated by epigenetic modifications during tumorigenesis 41, 42. In addition, according to our results in this report, the dynamic expression of miRNAs at different time points strongly suggests the involvement of epigenetic modification in regulating miRNA expression. As a result, it is worthy of paying attention to interactions among the epigenetic modification, miRNA and related neural targets during cortical interneuron in the future study.

Taken together, the current study shows that miRNA could be divided into several distinct clusters based on the global miRNA‐seq data and specific miRNA in each cluster might play distinct role at different stages of cortical interneural differentiation.

This *in vitro* differentiation model provides a robust platform for recapitulating cortical interneuron development process and can be used as a useful model to identify possible key miRNA regulators. Furthermore, the specific miRNAs that are identified in different differentiation stages could be considered as a potential biomarker or drug target of neurodegenerative disorders. This work also supports the perspective view that *in vitro* neuronal differentiation can provide a robust platform for neuronal disease modeling and related drug screening *in vitro* and potential cell replacement therapy in future clinical applications.

## Author contributions

JT, DDC, LL and HHC participated in carrying out the experiment and writing up the manuscript. HHC and WYC participated in project design. All authors read and approved the final manuscript.

## Supporting information


**Fig. S1.** (A) Neural rosette‐like structure was formed at ~ 10 days after neural induction; (B) Spontaneous firing was detected at day 80 during differentiation MEAs; (C) miRNA TPM distribution for the differentiation samples; (D) Clustering dendrogram for the differentiation samples. Scale bar: 125 μm; (E) TSI distribution of all detected miRNAs.

**Fig. S2.** Dynamic expression (TPM) for (A) hsa‐let‐7 family, (B) hsa‐miR‐302 family, (C) hsa‐miR‐9 and (D) has‐miR‐124 during cortical interneuron differentiation from iPSCs.

**Table S1.** Expression of all miRNAs determined by RNA‐seq at different time points (D0, D11, D25 and D80). TSI for each miRNA was shown. The lists of miRNA with TPM >= 10, miRNA family, and 5p/3p pairs, were shown separately.

**Table S2.** List of differentially expressed miRNAs (DEGs). Filtered downregulated/upregulated DEGs and their predicted mRNA targets were also shown in separate worksheets.

**Table S3.** KEGG and GO analyses for the upregulated and downregulated genes.

**Table S4.** Predicted upstream miRNAs that potentially target genes known to be involved in cortical interneuronal differentiation. TPM for each miRNA was shown.
