# Supplementary figures and images for: MicroRNA profiling during directed differentiation of cortical interneurons from human‐induced pluripotent stem cells
Source: FEBS Open Bio. 2018 Feb 17;8(4):502–12. doi: 10.1002/2211-5463.12377 (PMC5881541; doi:10.1002/2211-5463.12377)

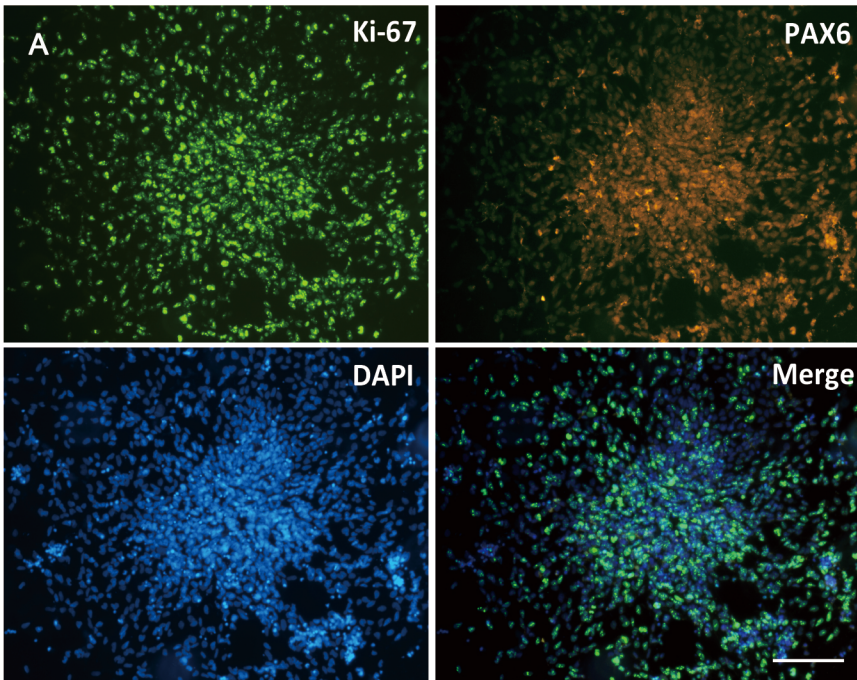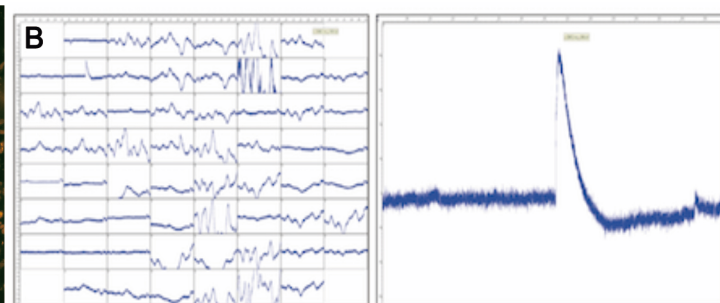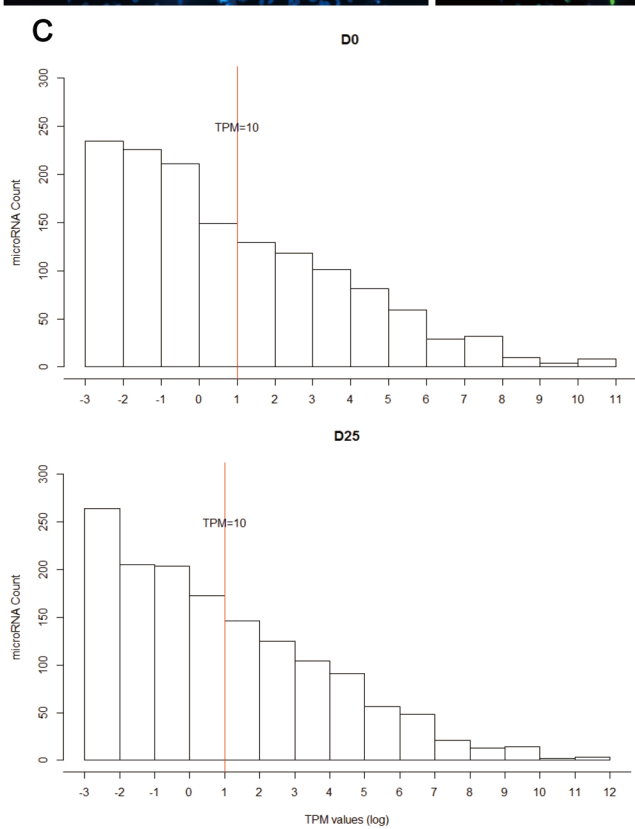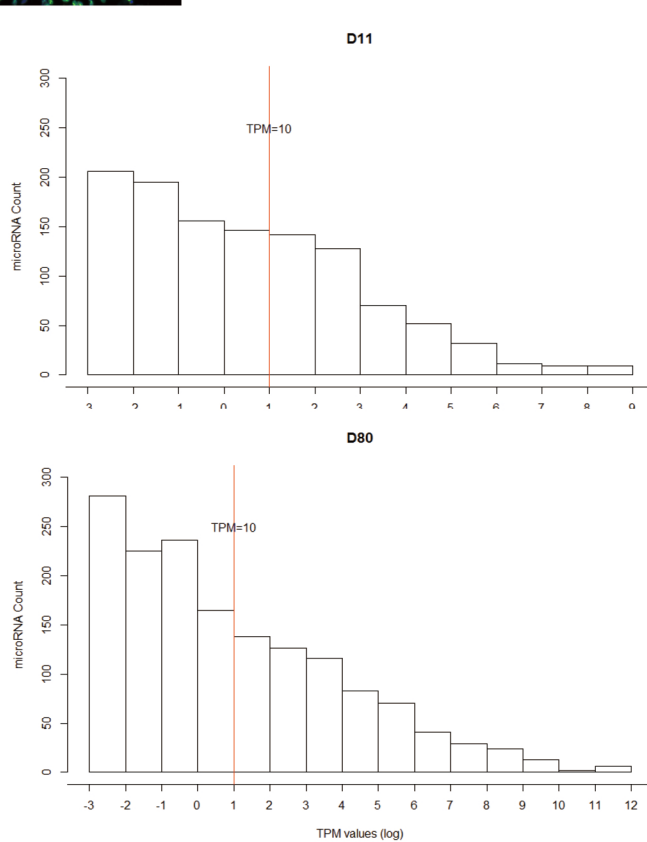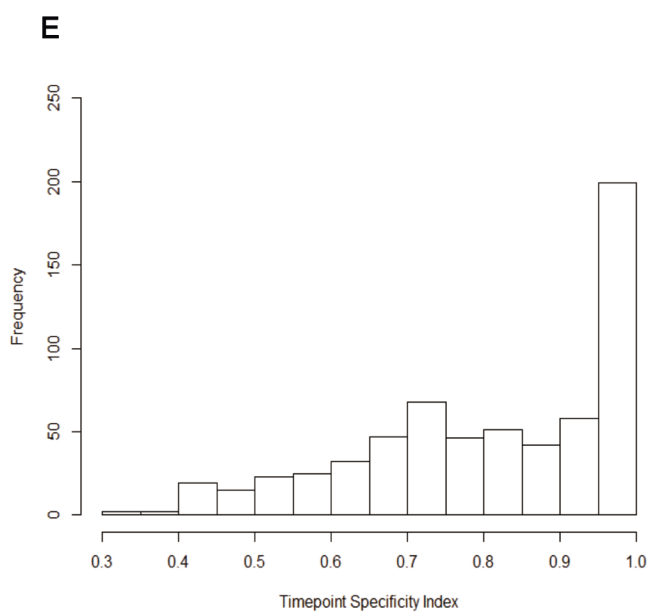

Supplement: Supplementary file 1 — Fig. S1. (A) Neural rosette‐like structure was formed at ~ 10 days after neural induction; (B) Spontaneous firing was detected at day 80 during differentiation MEAs; (C) miRNA TPM distribution for the differentiation samples; (D) Clustering dendrogram for the differentiation samples. Scale bar: 125 μm; (E) TSI distribution of all detected miRNAs. [file FEB4-8-502-s001.pdf]

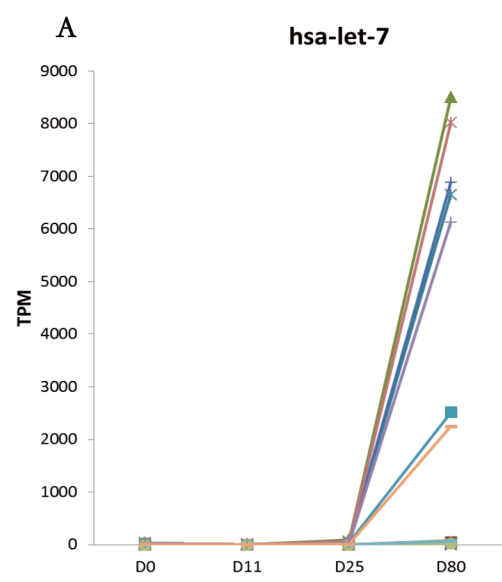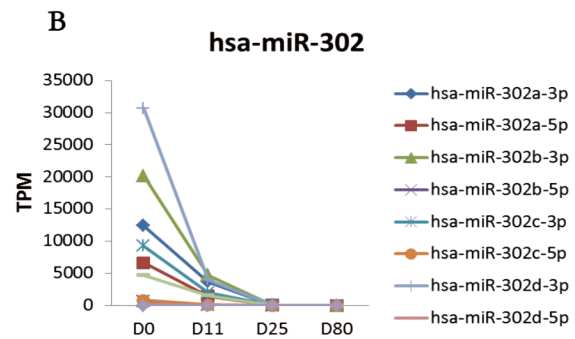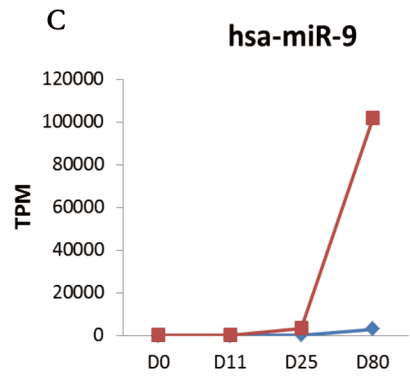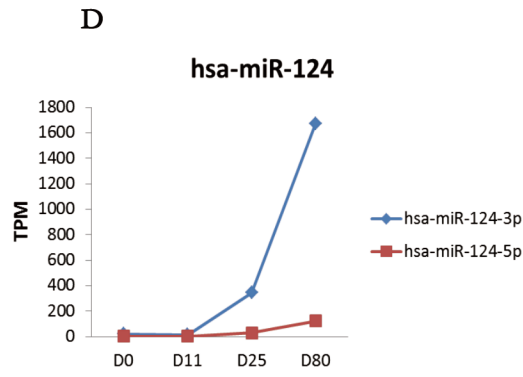

Supplement: Supplementary file 2 — Fig. S2. Dynamic expression (TPM) for (A) hsa‐let‐7 family, (B) hsa‐miR‐302 family, (C) hsa‐miR‐9 and (D) has‐miR‐124 during cortical interneuron differentiation from iPSCs. [file FEB4-8-502-s002.pdf]
